# Regulation of polysaccharide in Wu‐tou decoction on intestinal microflora and pharmacokinetics of small molecular compounds in AIA rats

**DOI:** 10.1186/s13020-024-00878-1

**Published:** 2024-01-13

**Authors:** Di Yang, Xiaoxu Cheng, Meiling Fan, Dong Xie, Zhiqiang Liu, Fei Zheng, Yulin Dai, Zifeng Pi, Hao Yue

**Affiliations:** 1 Changchun University of Chinese Medicine, No. 1035 Boshuo Rd, Nanguan District, Changchun, 130117 China; 2 Jiangzhong Pharmaceutical Co, Ltd., Nanchang, 330000 China; 3 National Center of Mass Spectrometry in Changchun and Jilin Provincal Key Laboratory of Chinese Medicine Chemistry and Mass Spectrometry, Changchun Institute of Applied Chemistry, Chinese Academy of Sciences, Changchun, 130022 China

**Keywords:** Wu‐tou decoction, Intestinal flora, Polysaccharides, Small molecule compounds, Pharmacokinetic properties

## Abstract

Wu-tou decoction (WTD), a traditional Chinese medicine prescription, is used to treat rheumatoid arthritis (RA). It works by controlling intestinal flora and its metabolites, which in turn modulates the inflammatory response and intestinal barrier function. Small molecular compounds (SM) and polysaccharides (PS) were the primary constituents of WTD extract. In this work, a model of adjuvant-induced arthritis (AIA) in rats was established and treated with WTD, SM, and PS, respectively. 16S rRNA gene sequencing was used to examine the regulatory impact of the various groups on the disturbance of the gut flora induced by RA. Further, since PS cannot be absorbed into the blood, the influence of PS on the absorption and metabolism of SM was studied by examining their pharmacokinetic (PK) parameters of 23 active components in SM by UPLC-MS/MS. WTD was found to be more effective than PS and SM in alleviating arthritis in AIA rats, which may be related to changes in gut flora. The PK properties of 13 active compounds were altered after PS intervene. Based on the findings, PS may be able to manage the disruption of intestinal microbiota, enhance the intestinal environment of model animals, and hence influence SM absorption and metabolism.

**Supplementary Information:**

The online version contains supplementary material available at 10.1186/s13020-024-00878-1.

## Introduction

Rheumatoid arthritis (RA) is an autoimmune disorder that is characterized by persistent inflammation of the joints and bones, resulting in severe synovitis and osteoporosis [1, 2]. In addition to pain, stiffness, edema, and loss of joint function, RA patients are more likely to develop cancer and cardiovascular disease. Enhancing the intestinal flora may help reduce RA symptoms because studies have revealed that nearly all RA patients have changed intestinal flora [3].

Numerous bacteria that are vital for immunological control, metabolism, illness prevention, and digestion reside in the human gut [4]. When the balance between beneficial bacteria and “harmful bacteria” is disrupted and the “harmful bacteria” become overabundant, this can often lead to the development of diseases. Consequently, restoring the disturbed intestinal flora is an effective approach to treating diseases. Furthermore, the gut is a vital “metabolic organ” of the body, influencing the metabolic processes of endogenous substances such as bile acid metabolism and tryptophan metabolism, as well as playing a key role in the metabolism of exogenous drug substances [5, 6]. As our knowledge of the intestinal flora has grown, the potential of the intestinal flora for drug metabolism has become increasingly apparent. The gut is a significant location of medication absorption. The drug remains in the gut after oral administration and interacts with the diverse gut flora. Gut bacteria can metabolize drugs by secreting glycosidases, reductases, and other metabolic enzymes, thus influencing drug absorption and efficacy [7].

Researchers have been paying more and more attention lately to the ability of polysaccharides used in traditional Chinese medicine (TCM) to control the flora in the intestine [8]. Although TCM polysaccharides are difficult for the body to digest, they may still have an impact through interacting with the flora in the intestine. Studies have demonstrated that TCM polysaccharides have a variety of pharmacological effects, including anti-inflammatory [9], antioxidant [10], and immunomodulatory [11]. Some TCM polysaccharides also have the ability to treat disorders by altering the composition of gut flora [12]. Therefore, it is important to consider the pharmacological action of TCM polysaccharides and their regulatory effects on the gut flora.

Wu-tou decoction (WTD) is a TCM recipe composed of Aconiti Radix Preparata, Ephedrae Herba, Glycyrrhiza Radix, Paeoniae Radix Alba, and Astragali Radix. It is a classical Chinese medicine recipe for treating RA. The WTD water extraction was roughly decomposed into small molecule compounds (SM) with low molecular weight and polysaccharides (PS). Many investigations on the effective ingredients and mechanisms of WTD in the treatment of RA had been conducted [13–16]. In our previous study, we examined the components of WTD absorbed in the blood [17, 18], and it was found that WTD could be effective in treating RA by modulating the composition of the intestinal flora [19]. However, there had been relatively few research on the involvement of polysaccharides in WTD because they cannot be absorbed into the bloodstream. In this study, the 16S rRNA gene sequencing technique was used to investigate the role of PS and SM in the regulation of intestinal flora in AIA rats. After that, the impact of PS intervention on the pharmacokinetic (PK) characteristics of SM was evaluated using UPLC-MS/MS analysis. The function of PS in the TCM formula was clarified by examining the effects of changes in the gut flora composition on the PK properties of SM.

## Materials and methods

### Chemicals and reagents

Complete Freund’s adjuvant (CFA) was purchased from Chondrex Inc. (Redmond, WA, USA). Methanol, formic acid, acetonitrile, and isopropanol (chromatographic grade) were purchased from Fisher Scientific (Loughborough, UK). Ultrapure water was prepared using the Milli-Q plus system (Milford, MA, USA). Other chemicals were of analytical grade.

Reference standards of ephedrine, methylephedrine, albiflorin, calycosin-7-glucoside, oxypaeoniflorin, paeoniflorin, liquiritin apiosid, liquiritin, isoliquiritin, liquiritigenin, calycosin, isoliquiritigenin, glycyrrhizic acid, formononetin, glycyrrhetinic acid, reserpine (IS), and naringin (IS) were purchased from the Chinese Authenticating Institute of Material and Biological Products (Beijing, China). Benzoylpaeoniflorin, benzoylaconine, benzoylmesaconine, benzoylhypaconine, neoline, songorine, talatizamine, and fuziline were purchased from Lanyuan Biological technology Co., Ltd. (Shanghai, China). The purities of all references were more than 98%.

### Preparation of WTD, SM, and PS

A total of 1400 g of crude herbs, comprising 200 g Aconiti Radix Preparata (ZCW), 300 g Ephedrae Herba (MH), 300 g Glycyrrhiza Radix (GC), 300 g Paeoniae Radix Alba (BS) and 300 g Astragali Radix (HQ) were immersed in water solvent for 1 h and then extracted twice by refluxing with 14 L and 11.2 L water for 1.5 h, respectively. The two extracts were combined. Subsequently, half of the WTD extract was concentrated to 1.5 g/mL and stored. The other half of the WTD extract was concentrated to 0.5 g/mL, 95% ethanol was added to make the ethanol concentration of the extract 70%, and alcohol precipitation was allowed to stand overnight. After centrifugation, the supernatant (SM) and the precipitated fraction (PS) were separated. The SM was concentrated to 1.5 g/mL of crude drug, while the PS was dissolved in water and concentrated to 1.5 g/mL of crude drug. They were kept separately frozen.

### Animals

The Animal Experiment Protocol listed below was approved by the Institutional Animal Care Committee of Jilin University Research Ethics Committee Guide (20190068) and performed according to Jilin Provincial Laboratory Animal Regulations. 42 male Sprague–Dawley (SD) rats (180–220 g) were purchased from Liaoning Changsheng Biotechnology Co., Ltd (Dalian, China). The animals were raised in an SPF grade, temperature-controlled (25 ± 1 ℃) and humidity-controlled (50% ± 5%) room at a 12 h light–dark cycle. Their food and water were provided to them free of charge. The animals fasted for 12 h before the start of the experiment.

### Drug administration

In the study of intestinal microflora, 30 SD rats were randomly divided into 5 groups (n = 6 for each group), namely the normal control group (NC group), model group (AIA group), WTD group, SM group and PS group, respectively. The model group, the WTD group, the SM group, and the PS group each received 0.1 mL of CFA in the left hind foot, whereas the NC group received 0.1 mL of saline in the same location. The AIA model was successfully established two weeks later. Rats in the WTD group were orally administrated WTD (9.8 g crude drug/kg/day) [19, 20], SM group rats received SM (9.8 g crude drug/kg/day), PS group rats were given PS (9.8 g crude drug/kg/day). The drug was given continuously for 30 days. Pathological sections, serum for biochemical component analysis, and colon contents for 16S rRNA sequencing analysis were all taken 1 h following the last treatment.

In the PK study, 12 SD rats were divided into model group (MG group) and model + polysaccharide group (MPS group). Rats in the MPS group were given PS (9.8 g crude drug/kg/day) by oral administration, while rats in the MG group received the same amount of saline. After continuous administration for 21 days, rats in both groups were given SM (39.2 g crude drug/kg/day), and two groups of rats were subjected to a PK study. The aim was to find out the effect of PS on the PK properties of SM components. Whole blood was collected in a 1.5 mL centrifuge tube containing heparin sodium before and 0.083, 0.25, 0.5, 0.75, 1, 2, 4, 6, 8, 12, 24,36 and 48 h after administration.

### 16S rRNA gene sequencing

The E.Z.N.A.^®^ soil DNA extraction kit (Norcross, GA,) was used for DNA extraction. The V3-V4 variable region was PCR-amplified using primers the quality of the DNA extraction was established, and the composition of the intestinal flora was determined by sequencing using a high-throughput sequencer Miseq. PCoA analysis was used to evaluate the difference in bacteria composition between the NC group, AIA group, WTD group, SM group and PS group. LEfSe analysis was used to determine the dominant flora of the different groups and to highlight the differences in flora.

### Pharmacodynamic analysis

The ELISA kit to detect the levels of TNF-α and IL-6 in rat serum according to the instructions. The ankle joints of the right hind feet of rats were fixed with 10% paraformaldehyde, and histological examination was conducted using hematoxylin–eosin (HE) staining.

### PK study

100 μL plasma was mixed with 10 μL internal standard solution (containing 150 ng/mL reserpine and 200 ng/mL naringin) and 500 μL isopropanol, shaken for 10 min. The supernatant was collected and dried with N_2_, redissolved with 100 μL methanol, and centrifuged again after 10 min centrifugation at 4 °C, 13000 rpm. Then the supernatant was collected for UPLC-MS /MS analysis.

### Instruments and analytical conditions

The Shimadzu UPLC-MS/MS system was used for the PK study. The system consists of high-performance liquid chromatography (LC-30A) and a triple quadrupole mass spectrometer (LCMS-8050) with ESI source. The multiple reaction monitoring (MRM) mode was used both in positive and negative mode. The MS parameters were as follows: flow rate of heating gas 10 L/min, flow rate of drying gas 10 L/min, flow rate of atomizing gas 3 L/min, interfacial temperature 300 ℃, DL temperature 250 ℃. The LabSolution LCMS ver. 5.6 software (Shimadzu, Japan) for data collection and processing.

Separation was performed using a Waters ACQUITY UPLC BEH C_18_ column (2.1 mm × 100 mm, 1.7 μm). The mobile phase was 0.1% formic acid water (v/v) (A) and acetonitrile (B). The optimal elution conditions were as follows: in positive ion mode, 0–8 min, 15–30% B; 8–10 min, 30–100% B; 10–12 min, 100% B; 12–14 min, 100–15% B; 14–17 min, 15% B. In negative ion mode, 0–2 min, 15–30% B; 2–7 min, 30–60% B; 7–8 min, 60–100% B; 8–10 min, 100% B; 10–12 min, 100–15% B; 12–15 min, 15% B.

### Statistical analysis

All data were shown as the mean ± SEM. GraphPad Prism version 8.0.1 (GraphPad Software, San Diego, USA) was used for statistical analyses. The significance of multiple groups was assessed using one-way analysis of variance (ANOVA) with Dunnett’s *post hoc* test. When data were not normally distributed, the Kruskal–Wallis H and Mann–Whitney U tests were performed. Significance was defined as *p* < 0.05 using SPSS 16.0 software. Compared with model *p < 0.05, **p < 0.01, ***p < 0.001.

## Results and discussion

### Pharmacodynamic evaluation

**Fig. 1:**
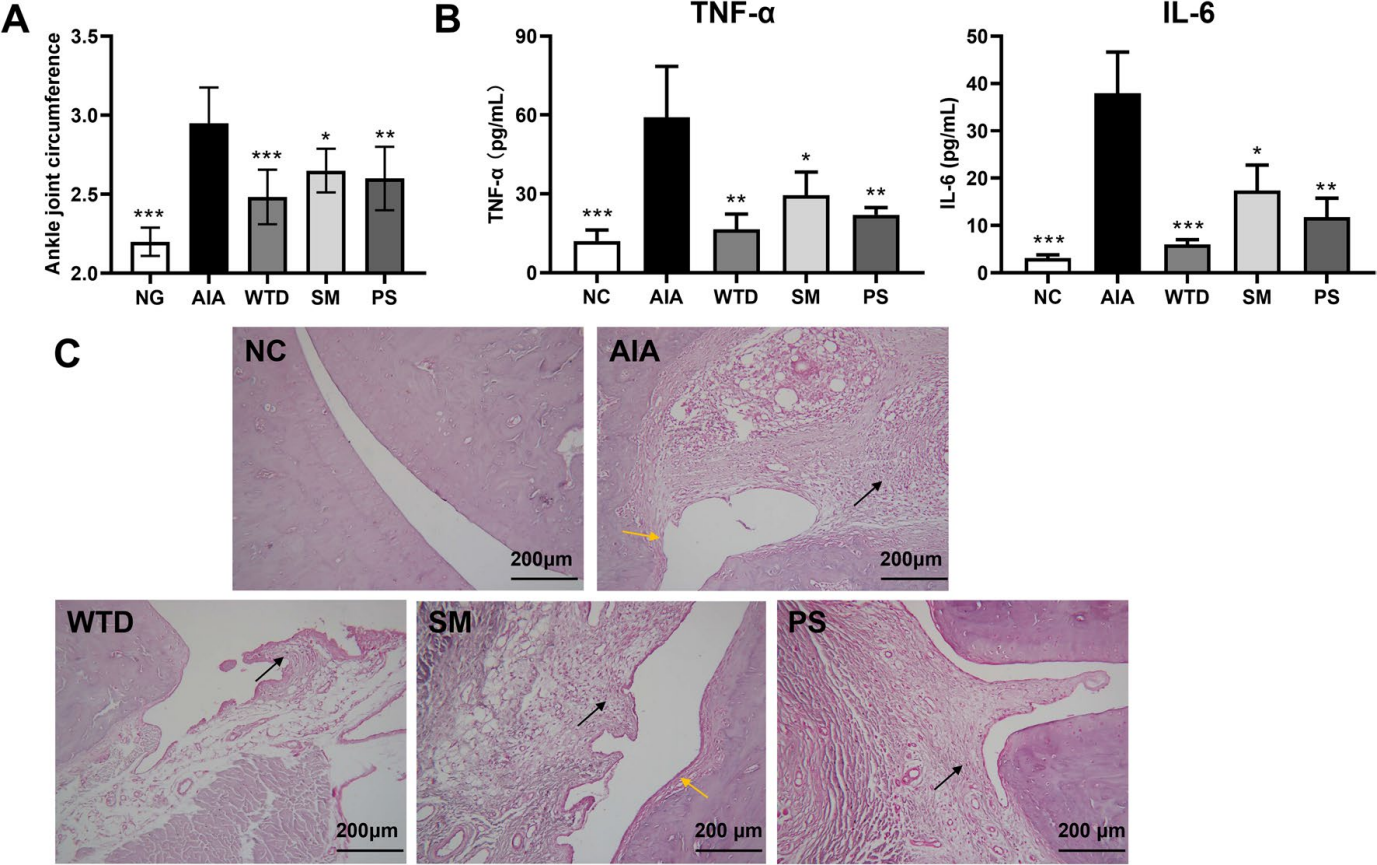
The levels of ankle swelling (**A**), levels of TNF-α and IL-6 in the serum of rats in each experimental group (**B**) and the pathological sections of the ankle joint of the right hind foot (**C**). Black arrow indicates inflammatory cell infiltration, and yellow arrow indicates bone and cartilage destruction and erosion. Results were expressed as mean ± SEM. *P* value was determined by one-way ANOVA with Dunnett’s* post hoc* test. Compared with model, *p < 0.05, **p < 0.01, ***p < 0.001 (n = 6)

In order to investigate the overall effect of WTD and different components, the pharmacodynamics of WTD (yield 52.12%/ crude drug), SM (yield 24.79%/ crude drug), and PS (yield 22.55%/ crude drug) were evaluated. Polysaccharide content in WTD, SM, and PS were determined by phenol–sulfuric acid method (Additional file 1: Table S1.). Their mass spectra were shown in Additional file 1: Figure S1. The ankle swelling diminished after drug administration (Fig. 1A). The levels of TNF-α and IL-6 in rat serum were measured using an ELISA kit, and the anti-inflammatory effects of the different groups were assessed. In addition, the HE staining procedure was used to study the pathology of the right hind foot’s ankle joint. The results showed that the AIA group had considerably greater levels of TNF-α and IL-6 in their serum than the NC group (Fig. 1B). The AIA rats' synovial tissue was badly proliferated, accompanied by a massive influx of inflammatory cells (Fig. 1C). WTD was able to successfully limit the inflammatory response both inside and outside of the joint, diminish the synovial destruction, and dramatically lower the levels of inflammatory factors in the serum. SM and PS were also able to inhibit the expression of inflammatory factors in the serum and alleviate the pathological changes of the joints induced by RA, though their effects were weaker than WTD.

### Analysis of gut microbiota composition

**Fig. 2:**
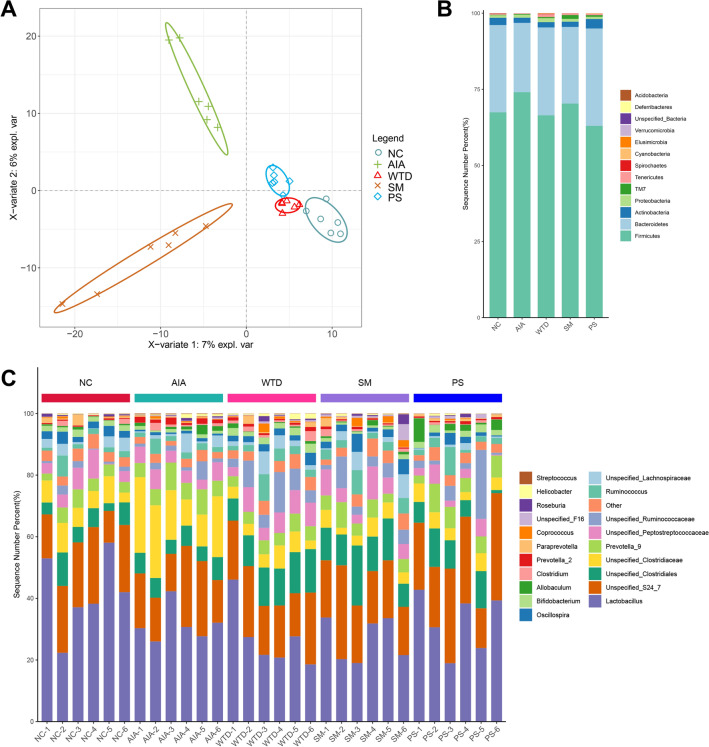
Modulatory effects of WTD, SM and PS on the composition of the intestinal flora. **A** PCoA analysis, **B** Average relative abundance of bacterial taxa at the phylum level, and **C** Relative abundance of bacterial taxa at the genus level

The 16S rRNA gene sequencing technology was used to examine the microbiota in rats' colon contents. The PCoA results demonstrated that the AIA group's intestinal flora was considerably different from the NC group, and the composition of the intestinal flora changed amongst the experimental groups (Fig. 2A). At the phylum level, AIA group had significantly lower abundance values for Bacteroidetes, Actinobacteria, and Tenericutes compared with the NC group. In contrast, Firmicutes abundance values increased. Compared with the AIA group, the relative abundance of Bacteroidetes and Tenericutes increased in the WTD, SM and PS groups, and the general trend was similar to the NC group (Fig. 2B). At the genus level, compared to the NC group, the AIA group showed an increase in the abundance values of Unspecified Clostridiaceae and Prevotella, while the abundances of Oscillospira and Bifidobacterium decreased. The relative abundance of Unspecified Clostridiaceae and Prevotella decreased and Oscillospira and Bifidobacterium increased in the administered groups by comparison with the AIA group (Fig. 2C). The above results indicate that RA could alter the composition of the intestinal flora in rats and cause disruption. WTD, SM, and PS had distinct restorative effects on the disordered gut bacterial composition.

### Differential microflora analysis

**Fig. 3:**
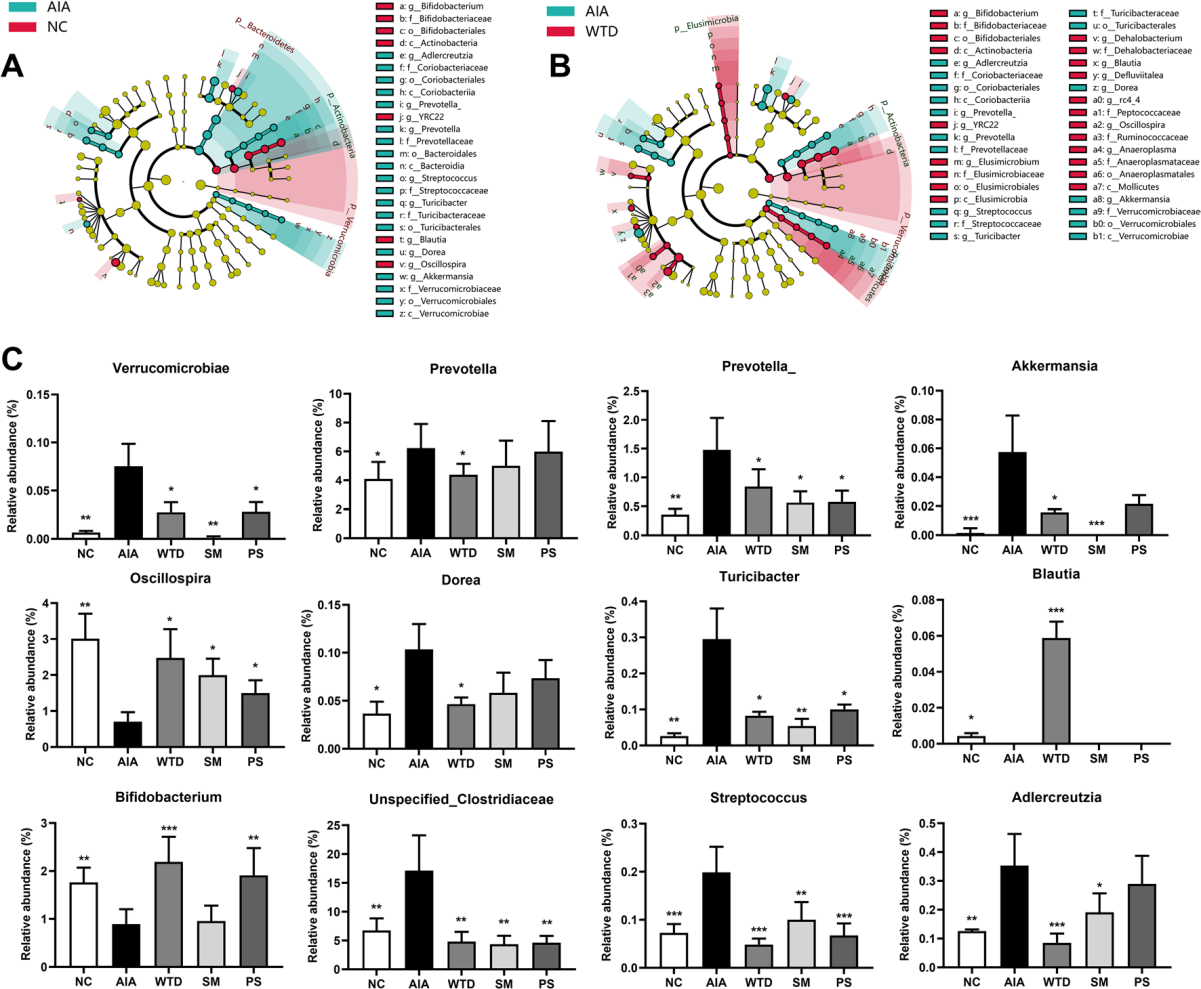
LEfSe analysis (**A** normal control (NC) vs model group (AIA), **B** AIA vs WTD group) and relative abundance of differential intestinal flora in each experimental group (**C**). Results were expressed as mean ± SEM. *P* value was determined by one-way ANOVA with Dunnett’s *post hoc* test. Compared with model, *p < 0.05, **p < 0.01, ***p < 0.001 (n = 6)

PCoA analysis demonstrated disparities in the composition of the intestinal flora among the experimental groups, thus the LEfSe analysis was utilized to identify the differential flora among these groups. The results of the LEfSe and PCoA analysis revealed a total of 12 key differential flora (Fig. 3).

When compared to the NC group, the colonic contents of AIA rats had considerably higher concentrations of Verrucomicrobiae, Akkermansia, Prevotella, Dorea, Turicibacter, Streptococcus, Adlercreutzia, and Unspecified_Clostridiaceae, with Akkermansia showing the greatest increase. Akkermansia is an intestinal mucin-degrading bacterium. Chiang et al. reported that the abundance of Verrucomicrobia and Akkermansia was significantly higher in RA patients compared to healthy individuals [21]. Additionally, some researchers have reported a significant increase in the abundance of Akkermansia in patients with juvenile spondyloarthritis, which was positively correlated with ankle swelling [22]. The elevated abundance of Akkermansia may be the primary cause of the elevated abundance of the Verrucomicrobia phylum. Prevotella abundance was also closely connected with RA, and patients with RA were frequently associated with elevated Prevotella abundance [23, 24]. Some Clostridium species in the Clostridiaceae family can produce toxins and cause bacterial illnesses. Individuals with RA, as well as those with both inflammatory bowel disease and arthropathy, had a higher abundance of Clostridiaceae [25]. Turicibacter is a pro-inflammatory bacterium that boosts the body's inflammatory response. It was also a pathogen found in increased concentrations in the guts of ulcerative colitis mice. Furthermore, Turicibacter abundance was positively correlated with the levels of TNF-α, IL-1β, and IL-6, suggesting that its overproduction may be linked to intestinal inflammation [26]. Streptococcus is a large group of common gram-positive cocci among septicococci, and some pathogenic streptococci can cause a variety of septic inflammatory diseases in humans.

In addition, there was a significant decrease in the abundance of Blautia, Bifidobacterium and Oscillospira in the colonic contents of rats in the model group. The abundance of Oscillospira was shown to be proportional to health and was significantly lower in patients suffering from inflammatory diseases [27]. Bifidobacterium is one of the most frequent probiotics in the human intestine and it is advantageous to human health because it performs a range of key physiological tasks such as biological barrier, nutritional impacts, immunomodulation, and gastrointestinal function enhancement. Blautia is a unique potential probiotic that has been shown in tests to reduce inflammation, promote SCFA synthesis, and maintain intestinal homeostasis in colorectal cancer patients [28]. The findings demonstrated that AIA rats had intestinal bacteria abnormalities, and that WTD, SM, and PS could all regulate the intestinal flora disorders produced by RA to varying degrees.

### Effects of PS on the PK profile of SM.

**Fig. 4:**
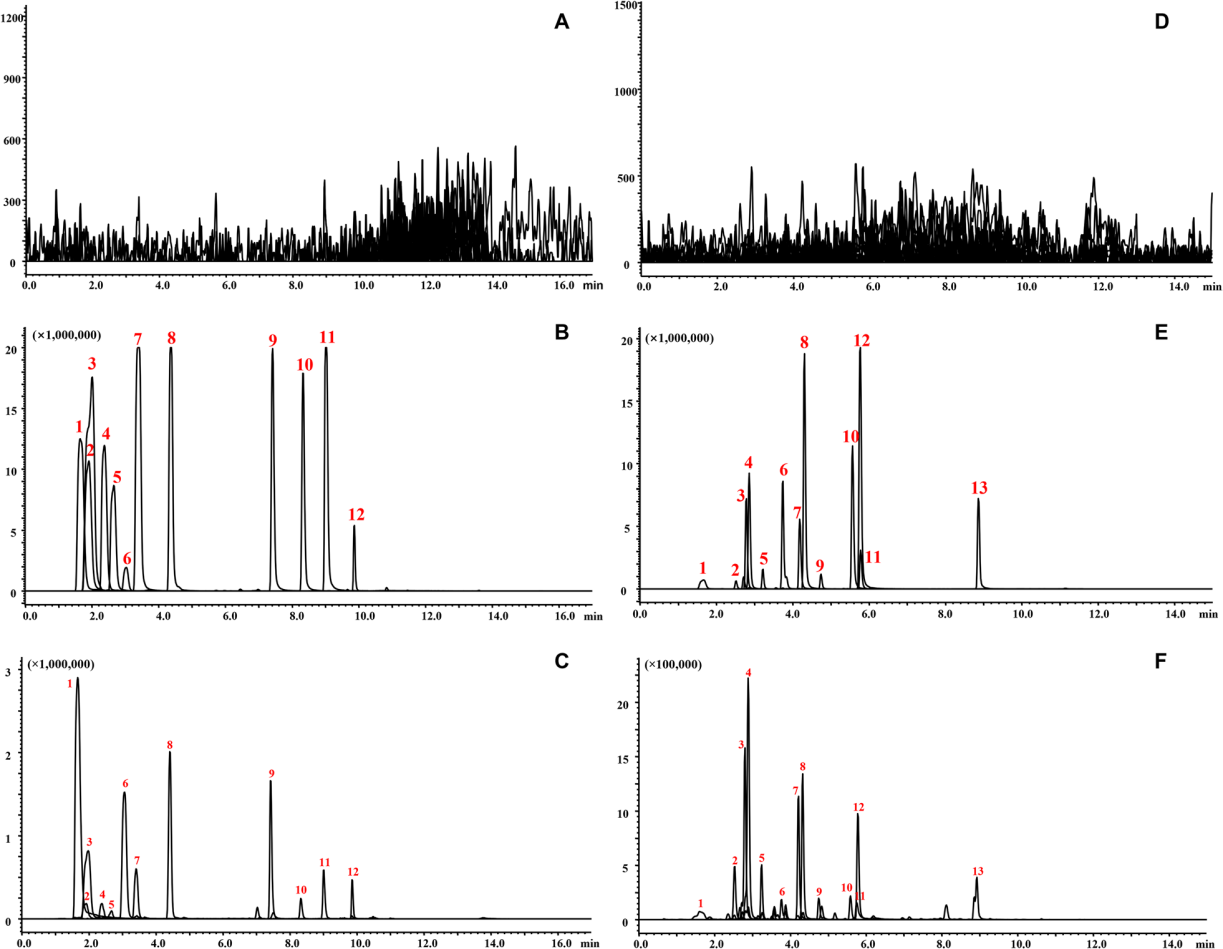
Representative MRM chromatograms of blank plasma, blank plasma containing standards and actual samples (0.25 h). Positive spectrum: **A**, **B** and **C**; negative spectrum: **D**, **E** and **F**. Positive spectrum: 1. Ephedrine, 2. Methylephedrine, 3. Songorine, 4. Fuziline, 5. Neoline, 6. Albiflorin, 7. Talatizamine, 8. Calycosin-7-glucoside, 9. Benzoylmesaconine, 10. Benzoylaconine, 11. Benzoylhypaconine, 12. Reserpine. Negative spectrum: 1. Oxypaeoniflora, 2. Paeoniflorin, 3. Liquiritin apioside, 4. Liquiritin, 5. Naringin, 6. Isoliquiritin, 7. Liquiritigenin, 8. Calycosin, 9. Benzoylpaeoniflorin, 10. Isoliquiritigenin, 11. Glycyrrhizic acid, 12. Formononetin, 13. Glycyrrhetinic acid

In our previous work, 74 chemical components were measured [29], and mass spectrometry was applied to the isolated SM and PS. The specific constituents were provided in Additional file 1: Table S2. The majority of the components in SM were small molecules, whereas the components in PS, which were primarily polysaccharide components, had a minimal SM content. In this study, the focus was to investigate the changes in PK properties of these absorbed components in plasma under PS influence using UPLC-MS/MS analysis, and then to clarify the effect of PS on the absorption of SM. A total of 23 components from WTD were subjected to PK studies. The MRM parameters, standard curves and representative MRM chromatograms of the compounds to be tested were shown in Tables 1, 2, and Fig. 4, respectively.

**Table 1:** MRM parameters for each compound and internal standard to be tested in the PK study

Compounds	Ion Mode	R_t_ (min)	Precursor (m/z)	Product ion (m/z)	Q1 (V)	Collision energy (V)	Q3 (V)
Ephedrine	+	1.7	166.2	148.2	14	15	15
Methylephedrine	+	1.9	180.1	162.2	11	15	30
Songorine	+	1.9	358.1	340.1	20	30	20
Fuziline	+	2.4	454.2	436.1	12	33	21
Neoline	+	2.6	438.2	420.2	10	30	21
Albiflorin	+	3.0	480.9	105.2	23	25	21
Talatizamine	+	3.3	422.3	390.2	15	30	29
Calycosin-7-glucoside	+	4.2	446.9	285.1	21	21	20
Benzoylmesaconine	+	7.3	590.1	105.1	28	53	20
Benzoylaconine	+	8.3	604.4	105.0	22	54	19
Benzoylhypaconine	+	8.9	574.2	542.2	20	36	20
Reserpine	+	10.1	609.3	195.1	22	37	21
Oxypaeoniflorin	−	1.7	495.1	137.2	23	30	12
Paeoniflorin	−	2.6	479.1	448.9	23	10	22
Liquiritin apioside	−	2.9	549.3	255.2	24	32	11
Liquiritin	−	3.0	417.2	255.1	18	22	11
Naringin	−	3.3	579.3	271.1	24	35	17
Isoliquiritin	−	3.8	417.2	255.1	18	19	11
Liquiritigenin	−	4.3	255.2	119.1	12	24	11
Calycosin	−	4.5	283.1	211.2	29	34	20
Benzoylpaeoniflorin	−	4.9	583.1	431.0	40	17	19
Isoliquiritigenin	−	5.8	255.3	119.1	16	24	11
Glycyrrhizic acid	−	5.9	821.6	351.0	20	40	11
Formononetin	−	6.0	267.0	223.2	13	32	21
Glycyrrhetinic acid	−	9.1	469.5	425.4	12	40	20

**Table 2:** Standard curves and linear ranges for each compound to be tested in PK studies

Analytes	Calibration curve	r^2^	Linear range (ng/mL)
Ephedrine	y = 0.8825x + 3.7555	0.9928	10–1000
Methylephedrine	y = 1.5091x + 0.0095	0.9945	0.1–25
Songorine	y = 2.1609x-0.0359	0.9905	0.1–50
Fuziline	y = 1.4974x-0.0152	0.9915	0.1–25
Neoline	y = 0.4646x-0.0020	0.9923	0.1–50
Albiflorin	y = 0.1032x + 0.0452	0.9916	5–500
Talatizamine	y = 1.9382x-0.0038	0.9928	0.1–50
Calycosin-7-glucoside	y = 0.1515x-0.0034	0.9936	0.1–100
Benzoylmesaconine	y = 1.0726x-0.0059	0.9929	0.1–100
Benzoylaconine	y = 0.7982x + 0.0047	0.9962	0.05–10
Benzoylhypaconine	y = 1.5379x-0.0135	0.9973	0.1–10
Oxypaeoniflorin	y = 0.9149x-0.0075	0.9995	0.5–50
Paeoniflorin	y = 0.0324x + 0.0288	0.9938	2.5–2500
Liquiritin apioside	y = 2.8756x-0.1369	0.9985	1–2500
Liquiritin	y = 4.1916x + 0.1199	0.9961	1–500
Isoliquiritin	y = 3.6659x-0.0129	0.9999	0.5–50
Liquiritigenin	y = 2.9112x + 0.0522	0.9990	0.5–100
Calycosin	y = 9.7214x-0.0172	0.9958	0.1–25
Benzoylpaeoniflorin	y = 0.0862x-0.0007	0.9992	1–50
Isoliquiritigenin	y = 6.5489x-0.0406	0.9993	0.5–50
Glycyrrhizic acid	y = 0.6790x-0.3716	0.9982	100–10000
Formononetin	y = 13.0676x + 0.0165	0.9974	0.5–50
Glycyrrhetinic acid	y = 3.2768x + 0.3358	0.9948	100–20000

**Fig. 5:**
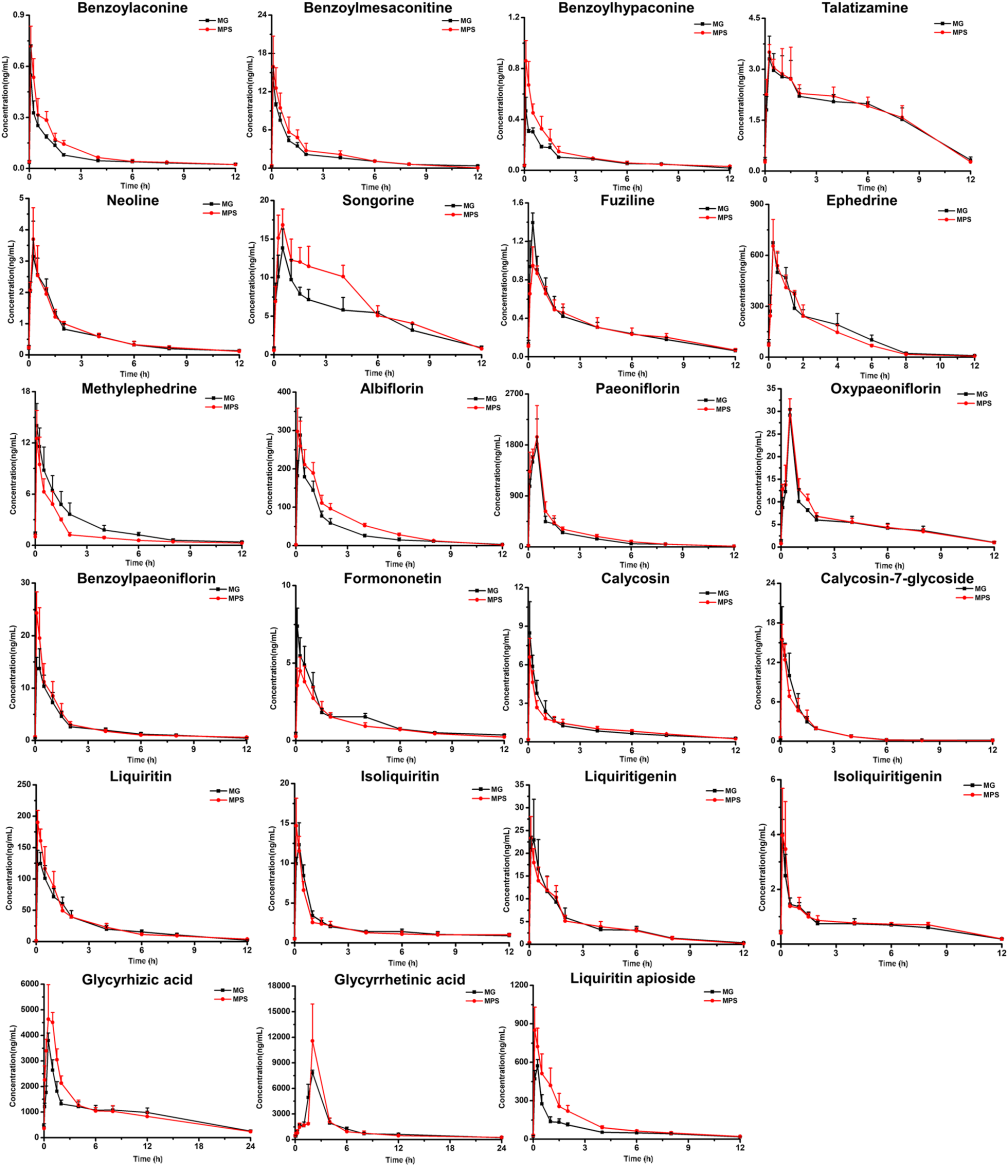
Mean blood concentration–time profiles of 23 potential pharmacodynamic substances (MG: model group, MPS: model + polysaccharide group)

The blood concentration–time curves of the 23 potential pharmacodynamic substances in each group were shown in Fig. 5. After the administration of polysaccharide, the songorine concentration–time curve changed significantly in the first 6 h. The C_max_ values of benzoylhypaconine, fuziline, oxypaeoniflorin, benzoylpaeoniflorin, formononetin, liquiritin, isoliquiritigenin, and glycyrhizic acid showed significant changes (*p* < 0.05). It shows that PS improves the absorption of active ingredients in AIA rats. Their PK parameters such as T _max_, C _max_, t _1/2_, AUC _0-∞_, and AUC _0-t_ were compared and analyzed (Table 3). They were calculated from PK Solver 2.0. Compared with the MG Group, after oral PS, the C _max_ and AUC _0-∞_ of benzoylhypaconine increased, AUC _0-t_ and AUC _0-∞_ were increased for songorine, paeoniflorin, albiflorin and liquiritin apioside. The C _max_ of liquiritin, benzoylpaeoniflorin, isoliquiritigenin and glycyrrhizic acid significantly increased. The C _max_, AUC _0-t_, and AUC _0-∞_ of oxypaeoniflora were all significantly higher. The C _max_ of fuziline and formononetin reduced, and the AUC _0-t_ and AUC _0-∞_ of methylephedrine decreased significantly. In summary, the PK characteristics of the components produced from GC and BS were primarily adjusted.

**Table 3:** PK parameters of 23 potential pharmacodynamic substances in the MG and MPS groups

Component name	Group	t _1/2_(h)	T _max_(h)	C _max_(ng/mL)	AUC _0-t_(ng/ml*h)	AUC _0-∞_(ng/ml*h)	Source
Benzoylaconine	MG	7.430 ± 5.148	0.125 ± 0.072	0.548 ± 0.178	0.764 ± 0.082	1.015 ± 0.158	ZCW
	MPS	3.875 ± 1.842	0.083 ± 0.001	0.720 ± 0.116	1.064 ± 0.136	1.202 ± 0.128	
Benzoylmesaconitine	MG	3.530 ± 0.873	0.139 ± 0.079	14.305 ± 3.689	21.413 ± 1.140	23.447 ± 1.422	ZCW
	MPS	1.723 ± 0.296	0.083 ± 0.001	15.899 ± 4.818	25.368 ± 7.147	25.465 ± 7.145	
Benzoylhypaconine	MG	3.938 ± 0.322	0.083 ± 0.001	0.467 ± 0.108	1.018 ± 0.049	1.123 ± 0.062	ZCW
	MPS	4.742 ± 0.904	0.083 ± 0.001	0.861 ± 0.160*	1.394 ± 0.255	1.604 ± 0.229*	
Talatizamine	MG	6.242 ± 1.838	0.417 ± 0.276	3.376 ± 0.595	21.996 ± 1.581	30.766 ± 4.871	ZCW
	MPS	5.163 ± 1.483	0.292 ± 0.093	3.576 ± 0.320	22.338 ± 1.786	28.106 ± 2.545	
Neoline	MG	3.245 ± 0.999	0.333 ± 0.118	3.221 ± 1.040	7.417 ± 0.718	8.058 ± 0.838	ZCW
	MPS	2.486 ± 0.349	0.250 ± 0.001	3.695 ± 1.005	7.559 ± 0.880	7.980 ± 0.757	
Songorine	MG	2.638 ± 0.461	0.667 ± 0.236	13.961 ± 2.318	60.186 ± 6.505	64.576 ± 6.039	ZCW
	MPS	2.522 ± 0.269	0.917 ± 0.773	17.665 ± 1.910	80.730 ± 5.544*	84.255 ± 5.364*	
Fuziline	MG	3.235 ± 0.929	0.250 ± 0.001	1.396 ± 0.100	3.636 ± 0.369	3.938 ± 0.411	ZCW
	MPS	3.196 ± 0.607	0.375 ± 0.125	1.032 ± 0.122*	3.590 ± 0.513	3.917 ± 0.539	
Ephedrine	MG	1.852 ± 0.273	0.250 ± 0.001	673.726 ± 137.169	1721.094 ± 96.813	1746.650 ± 93.580	MH
	MPS	1.736 ± 0.108	0.333 ± 0.118	661.523 ± 149.062	1559.029 ± 227.025	1575.759 ± 228.293	
Methylephedrine	MG	4.131 ± 1.363	0.083 ± 0.001	14.012 ± 2.615	27.900 ± 4.829	30.265 ± 3.815	MH
	MPS	4.331 ± 0.765	0.083 ± 0.001	12.523 ± 3.287	16.109 ± 2.770*	18.607 ± 4.409*	
Albiflorin	MG	2.488 ± 0.458	0.250 ± 0.001	287.831 ± 42.177	424.990 ± 55.691	458.506 ± 69.994	BS
	MPS	1.669 ± 0.080	0.139 ± 0.072	306.851 ± 57.880	644.268 ± 174.084*	698.946 ± 175.788*	
Paeoniflorin	MG	2.132 ± 0.102	0.438 ± 0.108	1924.153 ± 225.157	2541.326 ± 95.887	2455.492 ± 223.257	BS
	MPS	1.992 ± 0.126	0.396 ± 0.180	2063.985 ± 352.472	2870.390 ± 71.706**	2904.597 ± 68.691*	
Oxypaeoniflorin	MG	3.408 ± 0.586	0.437 ± 0.110	28.569 ± 0.784	61.034 ± 2.601	67.007 ± 3.218	BS
	MPS	3.399 ± 0.306	0.458 ± 0.093	31.514 ± 0.848*	70.355 ± 1.558*	75.327 ± 2.198*	
Benzoylpaeoniflorin	MG	3.588 ± 0.489	0.139 ± 0.079	14.781 ± 2.659	27.904 ± 5.404	30.566 ± 6.082	BS
	MPS	6.957 ± 2.271	0.083 ± 0.001	24.334 ± 3.995*	31.559 ± 6.741	37.891 ± 9.822	
Formononetin	MG	4.271 ± 0.406	0.083 ± 0.001	7.387 ± 1.162	15.278 ± 1.823	17.533 ± 1.685	HQ
	MPS	3.756 ± 0.557	0.250 ± 0.001	4.499 ± 0.902*	12.291 ± 1.983	13.592 ± 1.930	
Calycosin	MG	4.942 ± 0.315	0.083 ± 0.001	8.479 ± 2.434	12.296 ± 1.063	14.276 ± 1.185	HQ
	MPS	3.531 ± 0.666	0.083 ± 0.001	6.610 ± 1.443	12.353 ± 0.972	13.577 ± 0.642	
Calycosin-7-glycoside	MG	2.342 ± 0.959	0.110 ± 0.062	17.607 ± 2.640	17.473 ± 2.858	18.097 ± 2.629	HQ
	MPS	2.113 ± 0.779	0.083 ± 0.001	15.506 ± 2.308	15.987 ± 2.778	16.625 ± 2.476	
Liquiritin	MG	2.089 ± 0.132	0.167 ± 0.083	125.917 ± 18.484	300.440 ± 52.811	306.988 ± 53.933	GC
	MPS	3.301 ± 0.429	0.083 ± 0.001	190.023 ± 19.153*	321.663 ± 49.723	339.781 ± 52.584	
Isoliquiritin	MG	5.850 ± 2.022	0.194 ± 0.079	13.586 ± 1.061	23.305 ± 0.956	30.845 ± 2.926	GC
	MPS	7.608 ± 1.853	0.111 ± 0.062	14.210 ± 1.547	21.893 ± 1.756	33.055 ± 3.187	
Liquiritigenin	MG	1.841 ± 0.242	0.139 ± 0.079	25.519 ± 7.560	48.549 ± 6.497	49.404 ± 6.315	GC
	MPS	1.448 ± 0.025	0.083 ± 0.001	23.367 ± 4.695	47.195 ± 4.360	47.548 ± 4.374	
Isoliquiritigenin	MG	3.117 ± 0.571	0.083 ± 0.001	4.003 ± 0.545	8.764 ± 1.421	9.614 ± 1.601	GC
	MPS	3.871 ± 0.889	0.167 ± 0.083	5.421 ± 0.422*	9.467 ± 1.445	10.514 ± 1.272	
Glycyrrhizic acid	MG	7.605 ± 1.142	0.500 ± 0.001	3796.250 ± 303.817	23,076.672 ± 1971.518	25,908.272 ± 2650.703	GC
	MPS	7.254 ± 0.086	0.667 ± 0.236	5196.097 ± 705.783*	29,642.523 ± 2974.308	27,449.610 ± 2279.134	
Glycyrrhetinic acid	MG	10.666 ± 3.723	2.000 ± 0.001	7828.313 ± 282.922	28,800.324 ± 986.106	32,851.097 ± 2465.278	GC
	MPS	10.750 ± 1.956	2.000 ± 0.001	11,572.293 ± 3757.206	30,512.597 ± 6563.268	34,507.972 ± 6423.101	
Liquiritin apioside	MG	4.060 ± 0.589	0.250 ± 0.001	571.677 ± 49.414	941.302 ± 42.085	1053.558 ± 63.193	GC
	MPS	2.969 ± 0.328	0.083 ± 0.001	850.691 ± 179.709	1580.263 ± 242.037*	1669.308 ± 244.694*	

^*^p < 0.05, **p < 0.01, compared with the MG group

After oral administration, Chinese herbs remain in the intestinal tract and interact with a large number of abundant intestinal flora. The intestinal flora is also referred to as a crucial "metabolic organ" of the human body because it affects not only the metabolism of exogenous drug components that can have a big impact on drug absorption but also the metabolism of endogenous substances like bile acids (BAs) and tryptophan. Researchers have been playing a lot of attention lately to how intestinal flora affects drug metabolism, and an increasing number of studies have revealed that intestinal flora is crucial for PK and pharmacodynamics. Intestinal flora can affect the metabolism and absorption of drugs by secreting abundant enzymes such as glycosidases and reductases, which in turn affect the efficacy of the drug. Liquiritin, liquiritigenin, glycyrrhizic acid, liquiritin apioside, paeoniflorin, oxypaeoniflora, and benzoylpaeoniflorin were typical herbal components that could be significantly metabolized by the intestinal flora [30, 31]. For example, most of the glycyrrhizic acid would be converted to glycyrrhetinic acid in the intestine by the action of intestinal flora, thus continuing its medicinal effect. Liquiritin could be metabolized to liquiritigenin. Paeoniflorin could also be metabolized to albiflorin, albiflorinaglycone, and deacylate albiflorin [32]. SM prototypes also can alleviate inflammation by increasing absorption. Paeoniflorin [33], calycosin [34], liquiritin [35], liquiritigenin [36], and glycyrrhetinic acid [37], for example, exhibit a wide spectrum of anti-inflammatory and immunomodulatory actions and have been found to be useful in the treatment of RA. These chemical components will continue to be targeted in subsequent studies.

Previous research by our group demonstrated that WTD might improve intestinal flora dysbiosis and relieve the aberrant flora metabolite alterations caused by RA in AIA rats. Spearman correlation analysis revealed a close association between flora metabolites and intestinal flora. As a result, we hypothesized that a portion of the therapeutic impact of WTD on RA could be mediated by intestinal flora via modulation of the inflammatory response and intestinal barrier function. The integrity of the intestinal barrier is also linked to the development of inflammation and drug transfer [38].

**Fig. 6:**
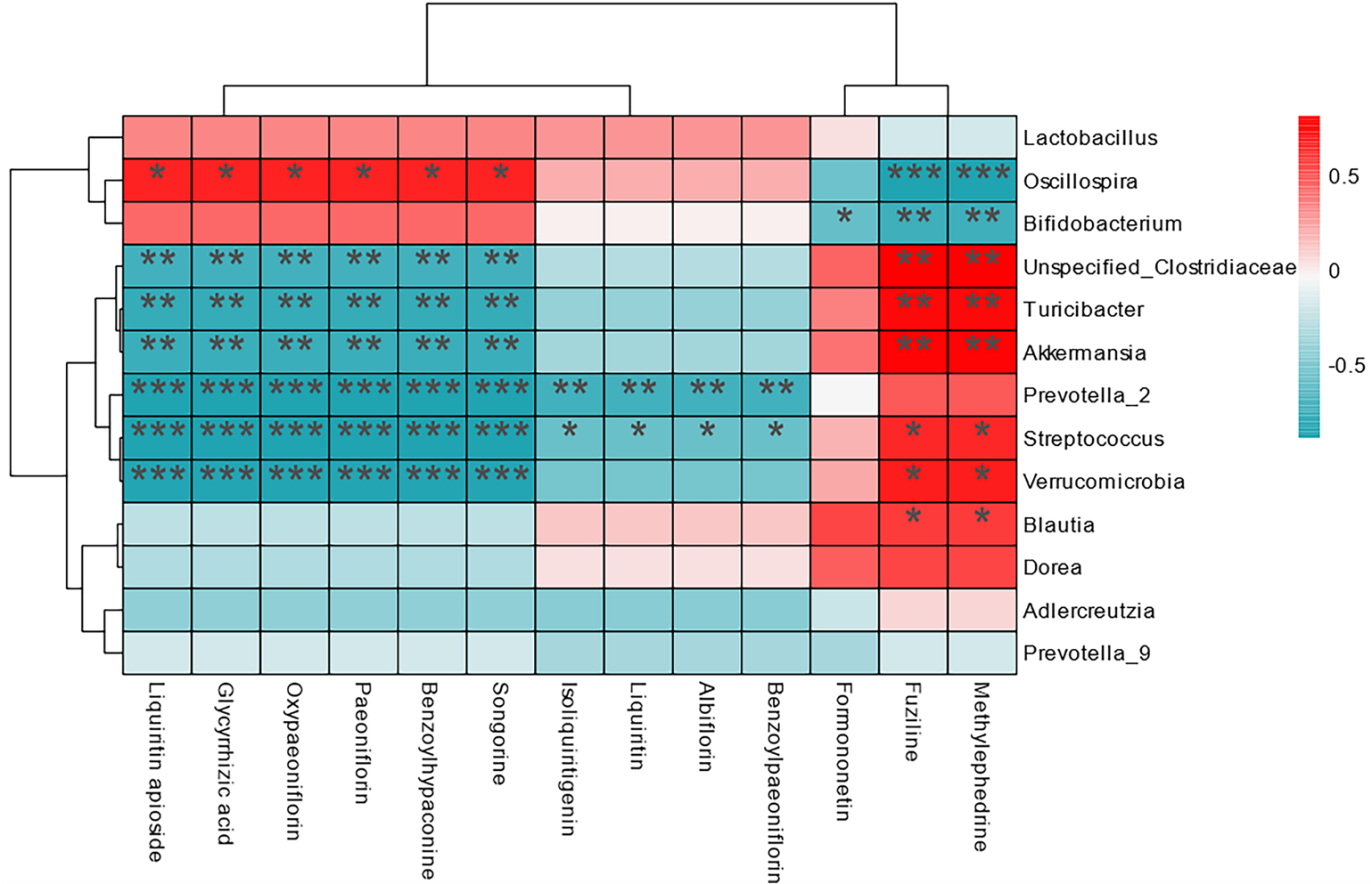
Correlation analysis between gut flora and SM

PS administration may modify the composition of intestinal flora in AIA rats, influencing SM absorption and metabolism. The association between SM absorption and gut flora was investigated by using Spearman correlation analysis. The differential flora impacted by the PS group was strongly linked with SM absorption, as shown in Fig. 6, with a total of 67 pairs had significant modifications. The drug absorption of liquiritin apioside, glycyrrhizic acid, oxypaeoniflorin, paeoniflorin, benzoylhypaconine, and songorine were negatively linked with Prevotella_2, meanwhile, whereas it exhibited a positive relationship with Oscillospira. Fuziline and methylephedrine exhibited a negatively correlation with Oscillospira and Bifidobacterium, while they were positively correlated with most of the differential flora. Furthermore, prior research had revealed that the intestinal barrier integrity of AIA rats has changed, and PS may also regulate the disruption of AIA rats' intestinal microbiota, repair the intestinal barrier of sick rats, and so affect SM absorption [19]. This could explain why the PK characteristics of the components in GC and BS had changed. Although polysaccharides cannot be absorbed into the bloodstream, they can influence the absorption and metabolism of small molecules by changing the gut bacteria, hence influencing medicinal efficacy. Products of microbiota metabolism can significantly mediate microbiota and host physiological function. Short-chain fatty acids (SCFAs), bile acids (BA), tryptophan metabolites and amino acids were important microbiota-associated metabolites that had immunomodulatory effects and were closely associated with RA disease progression [39]. In our preceding metabolomics studies, kynurenic acid, xanthurenic acid, tyrosine and phenylalanine were potential biomarkers that were associated with RA and WTD treatment. The changes in the content of a series of metabolites such as SCFA, BA, tryptophan metabolites and amino acids were further determined by targeted assays [17]. The results demonstrated that WTD can promote host health by alleviating metabolic disorders, reducing inflammation, modulating immune responses and maintaining intestinal barrier function. PS could affect the PK properties and absorption of pharmacodynamic substances in SM fraction in vivo. Hence, it was more logical to administer WTD as a combination of both PS and SM, which may result in improved pharmacodynamic effects.

It is imperative to recognize a noteworthy constraint: Firstly, the results of the study were not sufficient to demonstrate the role of gut microbiota affected by PS or SM on WTD effect. Secondly, PS from WTD could affect the PK of some SM to a certain extent. However, no correlation was established between PD and these components based on their changes in this study. In future studies, in-depth research should be conducted in these aspects to elucidate the underlying mechanisms of WTD more comprehensively.

## Conclusion

Drug metabolism and absorption are significantly influenced by intestinal flora, which has an effect on both drug toxicity and efficacy. In this study, the regulatory effects of WTD, SM, and PS fractions on the intestinal microbiota of AIA rats were clarified using 16S rRNA gene sequencing technology. WTD showed stronger efficiency than SM and PS in reducing RA-induced increased serum inflammatory factors, arthropathy, and intestinal flora problems.

Furthermore, UPLC-MS/MS analysis was used to further evaluate how PS intervention affected the PK profiles of SM. The PK profiles of 13 potential pharmacodynamic substances were altered under the intervention of PS. The absorption of benzoylhypaconine, songorine, paeoniflorin, albiflorin, liquiritin apioside, oxypaeoniflorin, liquiritin, benzoylpaeoniflorin, isoliquiritigenin, and glycyrrhizic acid were increased, while the absorption of fuziline, formononetin, and methylephedrine were decreased. GC and BS were the main sources of the components with changed PK profiles. As a result, PS may modify the composition of the intestinal flora of AIA rats, impacting the metabolism and absorption of SM. This allowed the absorbed SM in circulation to exercise their effects, which may explain why the efficacy of WTD was superior than SM and PS alone. As a result, it has been confirmed that the TCM recipe is more effective when taken as a whole.

### Supplementary Information

**Additional file 1: Table S1.** WTD extractions yield and Polysaccharide content. **Table S2.** Mass spectral data of compounds in SM and PS. **Figure S1.** Mass spectra of WTD, SM, and PS. (A, C, D were positive spectra for WTD, SM, and PS. B, D, F were negative spectra for WTD, SM, and PS.).

## Data Availability

Data available on request from the authors. The data that support the findings of this study are available from the corresponding author, Zifeng Pi, upon reasonable request.
