# Supplementary material for: Regulation of polysaccharide in Wu‐tou decoction on intestinal microflora and pharmacokinetics of small molecular compounds in AIA rats
Source: Chin Med. 2024 Jan 13;19:9. doi: 10.1186/s13020-024-00878-1 (PMC10787407; doi:10.1186/s13020-024-00878-1)
Supplement: Supplementary file 1 — Additional file 1: Table S1. WTD extractions yield and Polysaccharide content. Table S2. Mass spectral data of compounds in SM and PS. Figure S1. Mass spectra of WTD, SM, and PS. (A, C, D were positive spectra for WTD, SM, and PS. B, D, F were negative spectra for WTD, SM, and PS.). [file 13020_2024_878_MOESM1_ESM.docx]

**Supporting information**

**Regulation of polysaccharide in Wu‐tou decoction on intestinal microflora and pharmacokinetics of small molecular compounds in AIA rats**

Di Yang ^a#^, Xiaoxu Cheng^b#^, Meiling Fan^a^, Dong Xie^a^, Zhiqiang Liu^c^, Fei Zheng^a^, Yulin Dai^a^, Zifeng Pi^a^*, Hao Yue^a^*

*a Changchun University of Chinese Medicine, No.1035 Boshuo Rd, Nanguan District, Changchun, 130117, China*

*b Jiangzhong Pharmaceutical Co, Ltd., Nanchang 330000, China*

*c National Center of Mass Spectrometry in C**hangchun and Jilin Provincal Key Laboratory of Chinese Medicine Chemistry and Mass Spectrometry, Changchun Institute of Applied Chemistry, Chinese Academy of Sciences, Changchun, 130022, China*

^#^These two authors contributed equally to this paper.

*Corresponding authors:

Address: Changchun University of Chinese Medicine, No. 1035 Boshuo Rd, Nanguan District, Changchun, China.

Tel./Fax: +86 431 86172206 (Zifeng Pi), Tel./fax: +86 431 86172437 (Hao Yue).

E-mail addresses: 178521212@qq.com (Zifeng Pi), yuehao@ccucm.edu.cn (Hao Yue)

**Chemical composition analysis**

The analyzing methods of chemical composition were followed by the previous method. The phenol-sulfuric acid method[1] was applied to determine the total carbohydrate content with glucose as a standard. The yield of WTD (52.12%/ crude drug), SM (24.79%/ crude drug), and PS (22.55%/ crude drug) were evaluated. Polysaccharide content in WTD, SM, and PS were determined by phenol-sulfuric acid method (Table S1.)

**Components analysis of SM and PS**

74 chemical compounds had been found in WTD in previous studies[2], and the specific constituents were provided in Table S2. There were virtually no small molecule components in PS that were identical to SM (Figure 1S).

Table S1. WTD extractions yield and Polysaccharide content.

Table S2. Mass spectral data of compounds in SM and PS.

Figure S1. Mass spectra of WTD, SM, and PS. (A, C, D were positive spectra for WTD, SM, and PS. B, D, F were negative spectra for WTD, SM, and PS.)

Table S1. WTD extractions yield and Polysaccharide content

| Fraction | Yield (%) | Polysaccharide (%) |
| --- | --- | --- |
| Wu‐tou decoction (WTD) | 52.12 | 50.88±1.70 |
| Small molecular compounds (SM) | 24.79 | 8.21±0.90 |
| Polysaccharides (PS) | 22.55 | 78.89±0.89 |

Table S2. Mass spectral data of compounds in SM and PS.

| Peak No. | Indentification | Measured Mass | Ionization mode | Molecular formula | Error (ppm) | Source | WTD | SM | PS |
| --- | --- | --- | --- | --- | --- | --- | --- | --- | --- |
| 1 | Norephedrine or Norpseudoephedrine | 150.0906 | [M-H]^-^ | C_9_H_13_NO | -8.66 | MH | + | + | - |
| 2 | Ephedrine | 164.1061 | [M-H]^-^ | C_10_H_15_NO | -8.53 | MH | + | + | - |
| 3 | Gallic acid | 169.0128 | [M-H]^-^ | C_7_H_6_O_5_ | -5.33 | BS | + | + | - |
| 4 | Methylephedrine | 178.1235 | [M-H]^-^ | C_11_H_17_NO | 1.68 | MH | + | + | - |
| 5 | Liquiritigenin | 255.0673 | [M-H]^-^ | C_15_H_12_O_4_ | 6.27 | GC | + | + | - |
| 6 | Isoliquiritigenin | 257.081 | [M-H]^-^ | C_15_H_12_O_4_ | -1.56 | GC | + | + | - |
| 7 | Formononetin | 267.0642 | [M-H]^-^ | C_16_H_12_O_4_ | -5.62 | HQ | - | + | - |
| 8 | Calycosin | 283.0629 | [M-H]^-^ | C_16_H_12_O_5_ | 8.13 | HQ | + | + | + |
| 9 | Songorine | 356.2213 | [M-H]^-^ | C_22_H_31_NO_3_ | -3.65 | ZCW | + | + | - |
| 10 | Chuanfumine | 392.2423 | [M-H]^-^ | C_22_H_35_NO_5_ | -3.57 | ZCW | + | + | - |
| 11 | Isotalatizidine | 408.2764 | [M+H]^+^ | C_23_H_37_NO_5_ | 3.43 | ZCW | + | + | - |
| 12 | Liquiritin | 417.1148 | [M-H]^-^ | C_21_H_22_O_9_ | -9.11 | GC | + | + | + |
| 13 | Senbusine B | 422.2565 | [M-H]^-^ | C_23_H_37_NO_6_ | 5.21 | ZCW | + | + | - |
| 14 | Talatizamine | 422.2876 | [M+H]^+^ | C_24_H_39_NO_5_ | -7.10 | ZCW | + | - | - |
| 15 | Senbusine A | 424.2665 | [M+H]^+^ | C_23_H_37_NO_6_ | -8.01 | ZCW | + | + | - |
| 16 | 5-OH liquiritin | 433.1147 | [M-H]^-^ | C_21_H_22_O_10_ | 2.77 | GC | + | + | + |
| 17 | Neoline or Bullatine B | 438.2864 | [M+H]^+^ | C_24_H_39_NO_6_ | 1.83 | ZCW | + | - | - |
| 18 | Calycosin-7-glucoside | 447.1265 | [M+H]^+^ | C_22_H_22_O_10_ | -5.81 | HQ | + | + | - |
| 19 | Chasmanine | 452.3001 | [M+H]^+^ | C_25_H_41_NO_6_ | -2.43 | ZCW | + | + | - |
| 20 | Fuziline | 454.2793 | [M+H]^+^ | C_24_H_39_NO_7_ | -2.64 | ZCW | + | - | - |
| 21 | Hypaconine | 470.2713 | [M+H]^+^ | C_24_H_39_NO_8_ | -8.72 | ZCW | + | + | + |
| 22 | Glycyrrhetinic acid | 471.3467 | [M-H]^-^ | C_30_H_46_O_4_ | -1.49 | GC | + | + | - |
| 23 | Paeoniflorin | 479.1522 | [M-H]^-^ | C_23_H_28_O_11_ | -6.47 | BS | + | + | - |
| 24 | Pseudaconine | 484.292 | [M+H]^+^ | C_25_H_41_NO_8_ | 2.06 | ZCW | + | + | - |
| 25 | Mesaconine | 486.2697 | [M+H]^+^ | C_24_H_39_NO_9_ | -1.23 | ZCW | + | + | - |
| 26 | Oxypaeoniflorin | 497.1644 | [M-H]^-^ | C_23_H_28_O_12_ | -3.02 | BS | + | + | - |
| 27 | Aconitine | 500.2851 | [M+H]^+^ | C_25_H_41_NO_9_ | -1.80 | ZCW | + | + | - |
| 28 | Paeoniflorin sulfonate | 543.1188 | [M-H]^-^ | C_23_H_28_O_13_S | 2.95 | BS | - | + | - |
| 29 | Liquiritin apioside | 549.1643 | [M-H]^-^ | C_26_H_30_O_13_ | 6.37 | GC | - | + | + |
| 30 | Pyrohypaconitine | 556.2959 | [M+H]^+^ | C_31_H_41_NO_8_ | 8.81 | ZCW | + | - | - |
| 31 | Benzoyl-3,13-deoxymesaconine | 558.3073 | [M+H]^+^ | C_31_H_43_NO_8_ | 1.07 | ZCW | + | - | - |
| 32 | Benzoyl-3,13-deoxyaconine | 558.3089 | [M+H]^+^ | C_31_H_43_NO_8_ | 3.94 | ZCW | + | - | - |
| 33 | Pyromesaconitine | 572.2814 | [M+H]^+^ | C_31_H_41_NO_9_ | -8.04 | ZCW | + | + | - |
| 34 | Benzoylhypaconine | 574.3034 | [M+H]^+^ | C_31_H_43_NO_9_ | 3.13 | ZCW | + | + | - |
| 35 | Benzoylpaeoniflorin | 585.1934 | [M+H]^+^ | C_30_H_32_O_12_ | -6.49 | BS | + | + | - |
| 36 | Pyroaconitine | 586.2995 | [M+H]^+^ | C_32_H_43_NO_9_ | -3.58 | ZCW | + | + | - |
| 37 | Benzoyldeoxyaconine | 588.3124 | [M+H]^+^ | C_32_H_45_NO_9_ | -8.33 | ZCW | + | + | - |
| 38 | Benzoylmesaconine | 590.2961 | [M+H]^+^ | C_31_H_43_NO_10_ | -0.68 | ZCW | + | + | - |
| 39 | Benzoylaconine | 604.3104 | [M+H]^+^ | C_32_H_45_NO_10_ | -2.98 | ZCW | + | + | - |
| 40 | 10-OH benzoylmesaconine | 606.2901 | [M+H]^+^ | C_31_H_43_NO_11_ | -2.14 | ZCW | + | + | - |
| 41 | Hypaconitine | 616.3131 | [M+H]^+^ | C_33_H_45_NO_10_ | 1.46 | ZCW | + | - | - |
| 42 | 10-OH Benzoylaconine | 620.3072 | [M+H]^+^ | C_32_H_45_NO_11_ | 0.16 | ZCW | + | + | - |
| 43 | Deoxyaconitine | 630.3294 | [M+H]^+^ | C_34_H_47_NO_10_ | 2.54 | ZCW | + | + | - |
| 44 | Galloylpaeoniflorin | 631.1665 | [M-H]^-^ | C_30_H_32_O_15_ | 0.32 | BS | - | + | - |
| 45 | Mesaconitine | 632.3044 | [M+H]^+^ | C_33_H_45_NO_11_ | -4.27 | ZCW | + | - | - |
| 46 | Benzoylpaeoniflorin sulfonate | 647.1456 | [M-H]^-^ | C_30_H_32_O_14_S | 3.25 | BS | - | + | - |
| 47 | 10-OH mesaconitine | 648.3058 | [M+H]^+^ | C_33_H_45_NO_12_ | 5.86 | ZCW | + | + | - |
| 48 | 10-OH aconitine | 662.3189 | [M+H]^+^ | C_34_H_47_NO_12_ | 1.81 | ZCW | + | + | - |
| 49 | Glucoliquiritin apioside | 711.2176 | [M-H]^-^ | C_32_H_40_O_18_ | 5.62 | GC | - | + | - |
| 50 | Astragaloside IV | 783.4532 | [M-H]^-^ | C_41_H_68_O_14_ | 0.13 | HQ | + | + | - |
| 51 | Licorice saponin B2 | 807.4134 | [M-H]^-^ | C_42_H_64_O_15_ | -4.09 | GC | + | + | - |
| 52 | Licorice saponin E2 | 819.3764 | [M-H]^-^ | C_42_H_60_O_16_ | -4.76 | GC | + | + | - |
| 53 | Glycyrrhizic acid or Licorice saponin H2 or K2 | 821.3919 | [M-H]^-^ | C_42_H_62_O_16_ | -4.99 | GC | + | + | - |
| 54 | Astragaloside II | 827.4735 | [M+H]^+^ | C_43_H_70_O_15_ | -7.01 | HQ | + | - | - |
| 55 | 24-OH licorice saponin E2 | 835.3759 | [M-H]^-^ | C_42_H_60_O_17_ | 0.84 | GC | + | + | - |
| 56 | Licorice saponin G2 | 837.3917 | [M-H]^-^ | C_42_H_62_O_17_ | 0.96 | GC | + | + | - |
| 57 | 22-Acetoxyl licorice saponin C2 | 863.40 | [M-H]^-^ | C_44_H_64_O_17_ | -7.41 | GC | + | + | - |
| 58 | 22-Acetoxyl glycyrrhizic acid | 879.40 | [M-H]^-^ | C_44_H_64_O_18_ | 2.62 | GC | + | - | - |
| 59 | 22-Acetoxyl licorice saponin J2 | 881.41 | [M-H]^-^ | C_44_H_66_O_18_ | -2.61 | GC | + | + | - |
| 60 | Licorice saponin A3 | 983.4456 | [M-H]^-^ | C_48_H_72_O_21_ | -3.25 | GC | + | + | - |
| 61 | Licorice saponin J2 | 823.4108 | [M-H]^-^ | C_42_H_64_O_16_ | -0.97 | GC | + | + | - |
| 62 | 24-OH licorice saponin A3 | 999.4435 | [M-H]^-^ | C_48_H_72_O_22_ | -0.20 | GC | + | + | - |


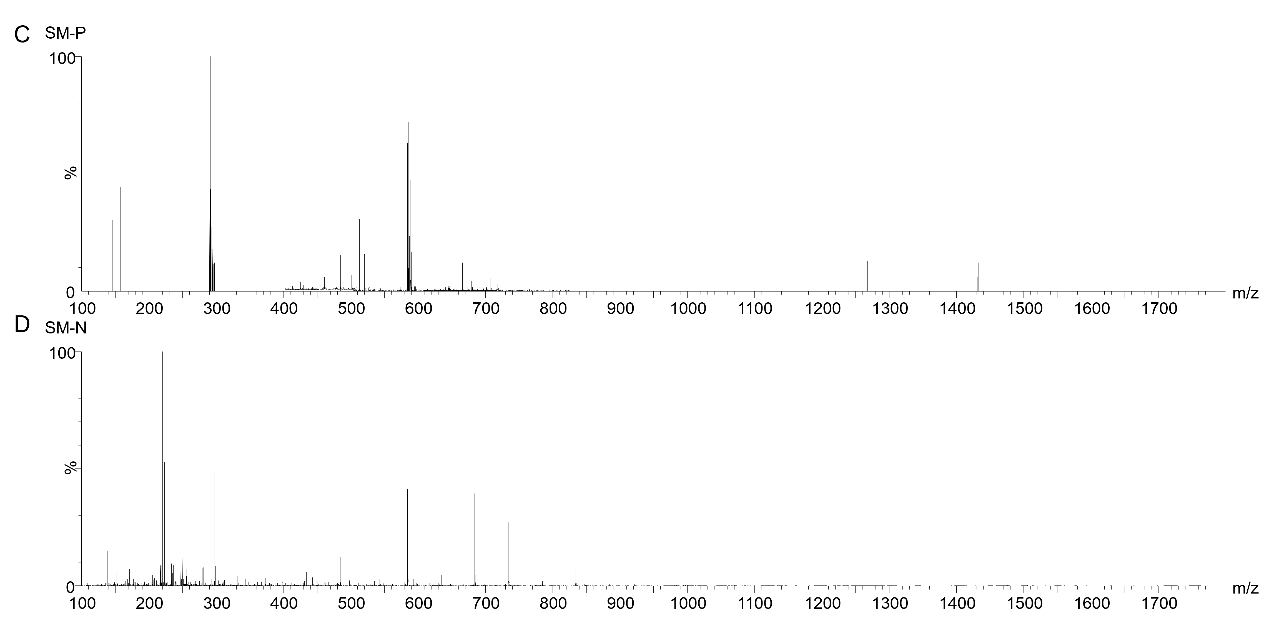

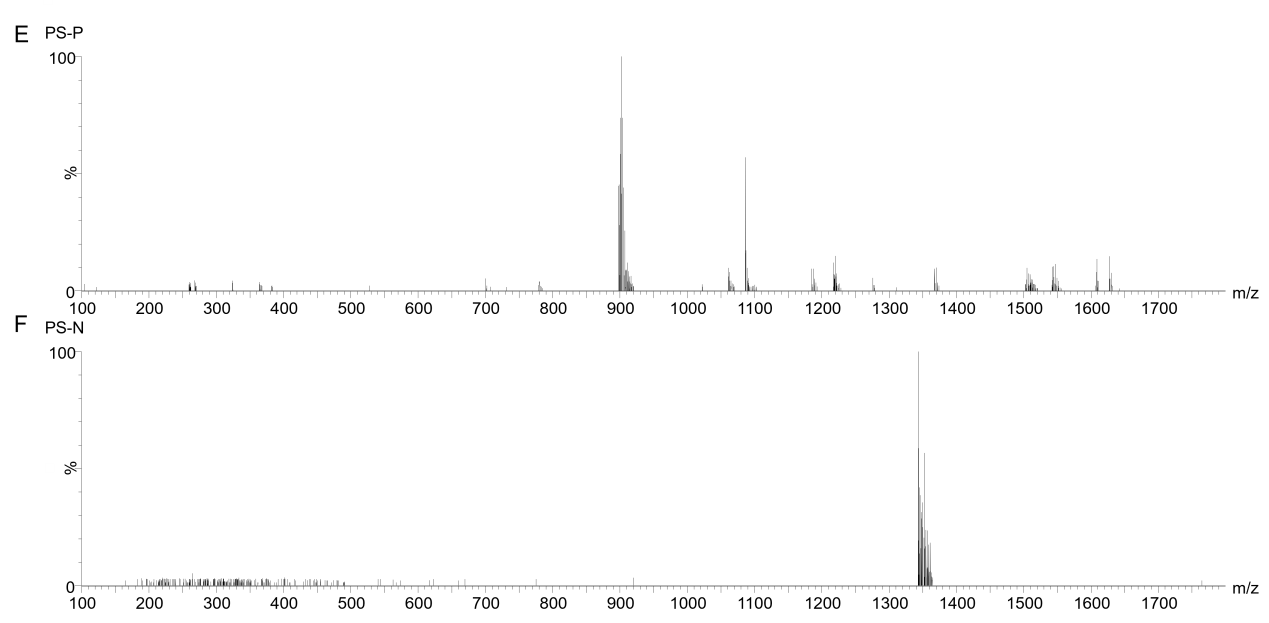

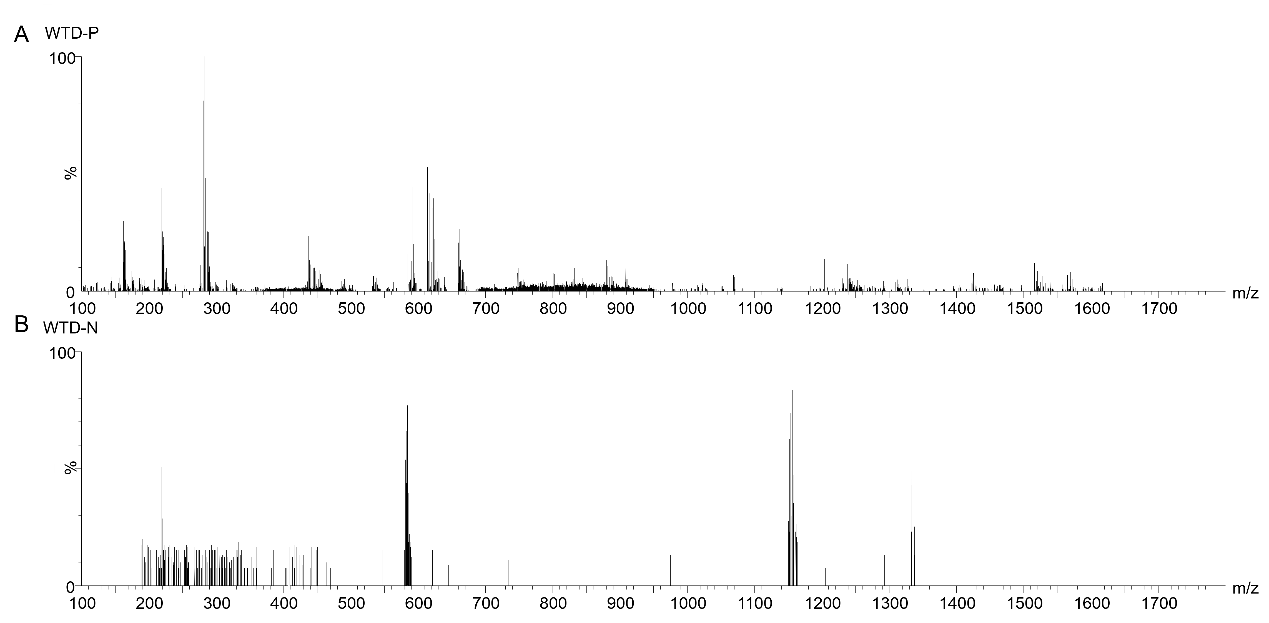


Figure S1. Mass spectra of WTD, SM, and PS. (A, C, D were positive spectra for WTD, SM, and PS. B, D, F were negative spectra for WTD, SM, and PS.)

1. M. Dubois, K.A. Gilles, J.K. Hamilton, P.A. Rebers, F. Smith, Colorimetric method for determination of sugar and related substances, Anal Chem. 1956; 28: 350-356.
2. Qi Y, Li S, Pi Z, Song F, Lin N, Liu S, Liu Z: Chemical profiling of Wu-tou decoction by UPLC-Q-TOF-MS. Talanta. 2014; 118:21-29.
